# FDG PET/CT for rectal carcinoma radiotherapy treatment planning: comparison of functional volume delineation algorithms and clinical challenges

**DOI:** 10.1120/jacmp.v15i5.4696

**Published:** 2014-09-08

**Authors:** Nadia Withofs, Claire Bernard, Catherine van der Rest, Philippe Martinive, Mathieu Hatt, Sebastien Jodogne, Dimitris Visvikis, John A. Lee, Philippe A. Coucke, Roland Hustinx

**Affiliations:** ^1^ Division of Nuclear Medicine and Oncological Imaging Department of Medical Physics, CHU of Liege Liege Belgium; ^2^ Division of Radiation Oncology Department of Medical Physics, CHU of Liege Liege Belgium; ^3^ Laboratoire de Traitement de l'Information Médicale (LaTIM) UMR 1101 Institut national de la santé et de la recherche médicale (INSERM) Brest France; ^4^ Department of Medical Physics CHU of Liege Liege Belgium; ^5^ Center of Molecular Imaging Radiotherapy, and Oncology (MIRO), Institut de Recherche Expérimentale et Clinique, Université catholique de Louvain Brussels Belgium

**Keywords:** FDG, PET, rectal cancer, segmentation, metabolic volume

## Abstract

PET/CT imaging could improve delineation of rectal carcinoma gross tumor volume (GTV) and reduce interobserver variability. The objective of this work was to compare various functional volume delineation algorithms. We enrolled 31 consecutive patients with locally advanced rectal carcinoma. The FDG PET/CT and the high dose CT (${\text{CT}}_{\text{RT}}$) were performed in the radiation treatment position. For each patient, the anatomical ${\text{GTV}}_{\text{RT}}$ was delineated based on the ${\text{CT}}_{\text{RT}}$ and compared to six different functional/metabolic ${\text{GTV}}_{\text{PET}}$ derived from two automatic segmentation approaches (FLAB and a gradient‐based method); a relative threshold (45% of the ${\text{SUV}}_{\text{max}}$) and an absolute threshold (SUV>2.5), using two different commercially available software (Philips EBW4 and Segami OASIS). The spatial sizes and shapes of all volumes were compared using the conformity index (CI). All the delineated metabolic tumor volumes (MTVs) were significantly different. The MTVs were as follows $\left(\text{mean}\pm \text{SD}):{\text{GTV}}_{\text{RT}}(40.6\pm 31.28\;\text{ml}\right)$; FLAB(21.36±16.34ml); the gradient‐based method (18.97±16.83ml); OASIS45%(15.89±12.68ml); Philips45%(14.52±10.91ml); OASIS2.5(41.62±33.26ml); Philips2.5(40±31.27ml). CI between these various volumes ranged from 0.40 to 0.90. The mean CI between the different MTVs and the ${\text{GTV}}_{\text{CT}}$ was <0.4. Finally, the DICOM transfer of MTVs led to additional volume variations. In conclusion, we observed large and statistically significant variations in tumor volume delineation according to the segmentation algorithms and the software products. The manipulation of PET/CT images and MTVs, such as the DICOM transfer to the Radiation Oncology Department, induced additional volume variations.

PACS number: 87.55.D‐

## I. INTRODUCTION

Radiation oncologists increasingly use ^18^F‐fuorodeoxyglucose (FDG) positron emission tomography (PET) during the essential step of gross tumor volume (GTV) delineation. Before incorporating PET in the treatment planning process in a clinical setting, there are a number of factors affecting image quantification and subsequent functional volume segmentation that need to be identified. The first determinants are related to tumor biology (e.g., heterogeneous glycolytic activity within the tumor) and location (e.g., in a region with high surrounding background activity like bladder).^1^, ^2^ Other factors are related to the physics principles of PET/CT acquisition and reconstruction protocols, which have all been identified as potential major factors.^3^, ^4^

Various (semi‐) automatic segmentation methods have been developed. To be clinically implemented, the ideal segmentation algorithm should be accurate, robust, and reproducible, as well as user‐friendly. The simplest algorithms are semiautomatic threshold‐based methods relying on the standardized uptake value (SUV) of each tumor voxel. These methods are heavily influenced by the intensity of tumor uptake so that various thresholds are applied, depending on individual situation, and regularly fail when the tumor is small or heterogeneous, or when the surrounding background activity is high.^2^, ^5^ More advanced threshold‐based segmentation methods take into account the background activity and the signal to background ratio to define the optimal threshold.^6^, ^7^ However, it has been also demonstrated that these adaptive threshold methods may fail to delineate small contrast or highly heterogeneous functional uptakes. Lastly, more advanced automatic algorithms based on image segmentation paradigms, such as gradient‐based methods^(8)^ and the fuzzy locally adaptive Bayesian algorithm (FLAB),^(9)^ have been proposed. In principle, the advantage of these mostly automatic algorithms is their robustness and reproducibility, taking into account varying imaging conditions such as lesion size, heterogeneity, or tumor uptake intensity in contrast with the surrounding background activity, as well as variable noise characteristics in the reconstructed PET images.^1^, ^10^, ^11^, ^12^

The primary objective of this work was to compare metabolic GTV delineation, based on FDG PET/CT images and derived from two different automatic segmentation approaches and from threshold‐based algorithms, using two commercially available software products. A secondary objective was to investigate volume variations related to the DICOM RT structure transfer between software products from a nuclear medicine to a radiation oncology department. This study focused on patients with rectal carcinoma. The FDG PET/CT is recommended for staging rectal carcinoma that usually displays high FDG uptake and provides a better interobserver agreement for rectal tumor delineation.^13^, ^14^, ^15^, ^16^

## II. MATERIALS AND METHODS

### A. Patients

We enrolled 31 consecutive patients with locally advanced rectal carcinoma (LARC) for which a FDG PET/CT was performed before chemoradiotherapy. The locoregional spread of the tumor was assessed using magnetic resonance imaging (MRI) and transrectal ultrasonography. The American Joint Committee on Cancer includes eight different T stages and nine N stages.^(17)^ We thus used a simplified approach and only distinguished the N0 from the N+ stage. The clinical stage was T2N+(N=3); T3N0(N=2); T3N+(N=23), or T4N+(N=3). Mean age of patients was 65±12yr. The retrospective analysis of the data was approved by the Ethics Committee of our Institution.

### B. FDG PET/CT

All 31 FDG PET/CT studies were performed on a Gemini TF PET/CT system (Philips Medical Systems, Cleveland, OH). Images were acquired 60 min after injection of 4 MBq/kg of FDG (220–440 MBq). All patients were positioned in the radiation treatment position on a flat pallet with the aid of a dedicated RT laser system placed in front of the PET/CT gantry. The low‐dose CT (5 mm slice thickness; tube voltage: 120 kV and tube current‐time product: 50 to 80 mAs, depending on the patient's weight) was followed by the PET emission scan with a time per bed position (pbp) depending on the patient's body mass index (BMI≤25: 1 min pbp; BMI≥26 and ≤32: 1 min 30 sec pbp; BMI≥33: 2 min pbp). Data were reconstructed using time of flight (TOF) information, as well as correction for decay, scatter, random, and attenuation (CT data were used for attenuation correction). The reconstructed CT matrix was 512×512 (voxel size $1.17\times 1.17\times 5\;{\text{mm}}^{3}$) and the PET matrix size was 144×144 (voxel size: $4\times 4\times 4\;{\text{mm}}^{3}$).

### C. CT simulation (${\text{CT}}_{\text{RT}}$)

All ${\text{CT}}_{\text{RT}}$ were performed in the Department of Radiation Oncology on a Big Bore system (Philips Medical Systems, Cleveland, OH) using standard clinical parameters (tube voltage: 120 kV and tube current‐time product: 250 mAs). For 21 of the 31 patients, the matrix size of the CT images was 1024×1024 (voxel size $0.59\times 0.59\times 3\;{\text{mm}}^{3}$). For 10 of the 31 patients, the matrix size of the CT images was 512×512 (voxel size $1.17\times 1.17\times 3\;{\text{mm}}^{3}$). The GTV (${\text{GTV}}_{\text{RT}}$) was delineated on the ${\text{CTR}}_{T}$ images by a radiation oncologist with a 12‐year experience in digestive oncology. He had also access to the FDG PET/CT and MR images. The median (range) delay between the FDG PET/CT and the ${\text{CT}}_{\text{RT}}$ was five days (1‐12 days).

### D. Image coregistration

For each patient, the low dose CT data and the ${\text{CT}}_{\text{RT}}$ data were automatically coregistered using the automatic rigid body registration tool called “local correlation” in the Extended Brilliance Workspace EBW‐NM 1.5.1 (EBW4, Philips Medical Systems). The same matrix transformation was applied to the FDG PET data. As most commercially available viewers resample the PET images into the CT, we applied a similar methodology for the coregistration process (e.g., all PET images were resampled onto the ${\text{CT}}_{\text{RT}}$ space leading to the same voxel size as the ${\text{CT}}_{\text{RT}}$).

### E. Segmentation methods

The coregistered FDG PET/CT data were used to delineate the metabolic tumor volumes (MTVs) using two software products: OASIS V1.8.3 (Segami Corporation, Columbia, MD) and EBW (Philips). In each case, the MTVs were delineated using two threshold‐based methods. One was based on a relative threshold, including all tumor voxels for which the activity reached 45% of the maximal SUV within the tumor. The other was based on an absolute threshold set at 2.5 SUV and including all tumor voxels for which the SUV was equal or superior to 2.5.^18^, ^19^ From here onwards, the volumes delineated using the OASIS software will be referred to as OA2.5 and OA45%, and the one delineated with the Philips EBW4 as PH2.5 and PH45%.

In addition, two segmentation algorithms were applied to automatically delineate the MTV: FLAB, taking into account image noise and limited spatial resolution through statistical and fuzzy modeling,^(9)^ and a gradient‐based method (GBM) relying on the watershed transform and cluster analysis for segmenting preprocessed PET images that are first denoised using an edge‐preserving filter and deblurred using iterative deconvolution.^(8^) Whatever the segmentation algorithm, all manipulations were performed by a nuclear medicine physician with 10 years of experience in oncological PET imaging, who identified the tumors and made sure no physiological uptake was included in the volumes delineated by the segmentation algorithms.

### F. Data analysis

The matrix sizes of the ${\text{CT}}_{\text{RT}}(1024\times 1024)$ and the low‐dose CT (512×512) of the PET/CT were different for 21 patients out of 31. For these 21 patients, we resampled the images of the PET and the low‐dose CT before applying any segmentation algorithm in order to match to the ${\text{CT}}_{\text{RT}}$ matrix size of 1024×1024. As a preliminary step in this subpopulation, we applied the different segmentation algorithms on both resampled and non‐resampled PET images for each 21 patients. We then compared the MTVs delineated according to whether the matrix had been resampled or not. We also compared the ${\text{SUV}}_{\text{max}}$ and ${\text{SUV}}_{\text{mean}}$ extracted from the volumes obtained with or without resampling.

We considered the GTV that was used for treating the patient (${\text{GTV}}_{\text{RT}}$) as the reference.

In order to visualize the spatial distribution of all delineated MTVs in a single PET image, we exported all MTVs and the ${\text{GTV}}_{\text{RT}}$ in the PMOD software (version 3.207; PMOD Technologies Ltd., Zurich, Switzerland). To estimate if the spatial distribution of the different MTVs were concordant, the conformity index (CI) was calculated between each MTV two by two, and between each MTV and the ${\text{GTV}}_{\text{RT}}$. The CI was calculated by dividing the intersection volume by the conjunction volume.^(20)^

Lastly, we transferred MTVs from OASIS and Philips Brilliance software products to Pinnacle^3^ software (Philips Medical Systems) in the Radiation Oncology Department. Each volume was transferred to Pinnacle and PMOD as a DICOM RT structure set via a network connection. We compared the transferred volumes to the initial volumes displayed in OASIS or Philips in order to estimate if the DICOM transfer to Pinnacle or PMOD had an impact on the delineated volume.

### G. Statistical analyses

In the 21/31 patients with a ${\text{CT}}_{\text{RT}}$ matrix size of 1024×1024, the Student's *t*‐test and Wilcoxon signed‐rank test were performed to estimate if the resampling of the PET images had an impact on the delineated MTVs and SUV measurements. In the next step, we compared the MTVs delineated in all the 31 subjects using the ANOVA‐2 or Friedman tests. When the ANOVA‐2 test was significant, a post‐hoc test (Scheffé test) was performed to compare volumes derived from the different segmentation algorithms two by two. Results were considered to be significant at the 5% level (p<0.05). Calculations were done using SAS version 9.2 (SAS Institute, Cary, NC).

## III. RESULTS

**A. Impact of PET images resampling on metabolic tumor volumes and SUVs**

**Figure 1:**
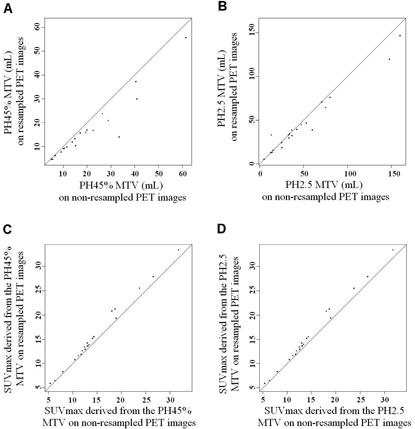
The scatter‐plots (a) and (b) show the metabolic tumor volumes (MTV) without resampling PET images (x‐axis) plotted against the MTV (mL) delineated on resampled PET images (y‐axis) using the Philips EBW4 software and a $45\%{\text{SUV}}_{\text{max}}$ threshold (a) or the SUV2.5 threshold (b). The scatter‐plots (c) and (d) show the ${\text{SUV}}_{\text{max}}$ extracted from the volumes delineated without resampling PET images (x‐axis) plotted against the ${\text{SUV}}_{\text{max}}$ extracted from the volumes delineated on resampled PET images (y‐axis) using the Philips EBW4 software and a $45\%{\text{SUV}}_{\text{max}}$ threshold (c) or the SUV2.5 threshold (d). The resampling of PET images significantly modified the volume delineated in the Philips EBW4 workspace and the extracted ${\text{SUV}}_{\text{max}}$ whatever the threshold‐based method used (PH45% or PH2.5).

The resampling of PET images significantly decreased the volumes delineated in the Philips EBW4 workspace (Figs. 1(a) and (b)). The mean^(21)^ relative variation of the delineated MTV was 26.9% (28.5%) for the PH45% and 7.39% (20.90%) for the PH2.5. The ${\text{SUV}}_{\text{max}}$ and ${\text{SUV}}_{\text{mean}}$ extracted from the delineated volumes using the Philips EBW4 workspace were significantly higher on resampled PET images than the SUV recorded on non‐resampled PET images, using both threshold‐based (PH45%; PH2.5) methods (Figs. 1(c) and (d)). On the other hand, the MTVs delineated in OASIS (OA45% and OA2.5) and with the FLAB method were not statistically modified by the resampling.

### B. Impact of the segmentation algorithm on the volumes and SUVs

**Figure 2:**
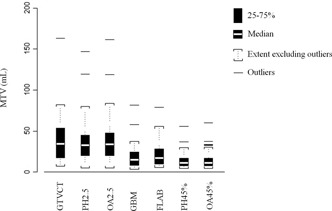
Box‐Whisker plot of tumor metabolic tumor volumes (MTV) delineated using threshold‐based methods ($45\%{\text{SUV}}_{\text{max}}$ or SUV 2.5) using two different software products (OASIS, Philips EBW4) and automatic segmentation algorithms using the GBM validated by Geets et al.^(8)^ or a FLAB segmentation approach.

The delineated volumes obtained in the 31 patients are presented in Fig. 2. Taken together, the MTVs were significantly different between all approaches (p<0.0001). All MTVs were significantly smaller than the ${\text{GTV}}_{\text{CT}}$, except those defined with the SUV2.5 threshold‐based methods (PH2.5 and OA2.5). There were no significant differences between the $45\%{\text{SUV}}_{\text{max}}$ threshold (PH45% and OA45%) and the automatic algorithms (gradient‐based and FLAB methods), between PH2.5 and OA2.5 or between PH45% and OA45%. Finally, the gradient‐based and FLAB volumes were not significantly different.

The MTVs delineated using the same threshold (SUV2.5 or $45\%{\text{SUV}}_{\text{max}})$ in two distinct software products (Philips EBW4 or OASIS) were the most consistent; the intraclass coefficients and the 95% confidence interval lower limits are presented in Table 1

We also found a significant difference in the spatial distribution of the different MTVs, even when the segmentation algorithm was identical but applied by two different software products (Philips EBW4 or OASIS). The conformity indices (CI) are presented in Table 2. The largest mean CI(≥0.87) was observed between the volumes delineated using an identical threshold‐based method (45%SUV or SUV2.5) applied in two different software products (OASIS and Philips EBW4). The CI between all other methods with one another and with the ${\text{GTV}}_{\text{CT}}$ were ≤0.70. The values of ${\text{SUV}}_{\text{mean}}$ extracted from the different MTVs were significantly different, but the ${\text{SUV}}_{\text{max}}$ were identical whatever the chosen algorithms (data not shown).

**Table 1:** Intraclass correlation coefficients (95% confidence interval lower limit) for all methods and two by two

	*All Methods*	${\text{GTV}}_{\text{RT}}$	*FLAB*	*OA45%*	*OA2.5*	*PH45%*	*PH2.5*	*GBM*
Overall	0.55							
	(0.38)							
${\text{GTV}}_{\text{RT}}$			0.50	0.31	0.85	0.26	0.84	0.54
			(0.09)	(‐0.02	(0.74)	(‐0.04	(0.72)	(0.02)
FLAB				0.86	0.49	0.80	0.52	0.91
				(0.51)	(0.09)	(0.31)	(0.10)	(0.83)
OA45%					0.33	0.95	0.36	0.80
					(‐0.02	(0.90)^a^	(‐0.01	(0.66)
OA2.5						(‐0.03	0.99	0.53
						(‐0.03	(0.98)^a^	(0.02)
PH45%							0.31	0.73
							(‐0.03	(0.53)
PH2.5								0.56
								(0.03)

a The metabolic tumor volumes delineated using the same threshold ($45\%{\text{SUV}}_{\text{max}}$ or SUV2.5) in two distinct software products (Philips EBW4 or OASIS) were the most consistent.

**Table 2:** Characteristics of the conformity indices between each metabolic tumor volume two by two

*Pair*	*N*	*Mean*	*SD*	*SE*	*Min*	*Max*	*Student's* t*‐test & Wicoxon test (H0:* Mean=1)
${\text{GTV}}_{\text{RT}}$ vs. FLAB	31	0.31	0.17	0.03	0.0	0.7	<0.0001
${\text{GTV}}_{\text{RT}}$ vs. OA45%	31	0.24	0.16	0.03	0.0	0.6	<0.0001
${\text{GTV}}_{\text{RT}}$ vs. OA2.5	31	0.36	0.21	0.04	0.0	0.7	<0.0001
${\text{GTV}}_{\text{RT}}$ vs. PH45%	31	0.24	0.14	0.03	0.0	0.6	<0.0001
${\text{GTV}}_{\text{RT}}$ vs. PH2.5	31	0.38	0.21	0.04	0.0	0.7	<0.0001
${\text{GTV}}_{\text{RT}}$ vs. GBM	31	0.25	0.15	0.03	0.0	0.6	<0.0001
FLAB vs. OA45%	31	0.69	0.13	0.02	0.4	0.9	<0.0001
FLAB vs. OA2.5	31	0.53	0.17	0.03	0.3	1.0	<0.0001
FLAB vs. PH45%	31	0.69	0.12	0.02	0.5	0.9	<0.0001
FLAB vs. PH2.5	31	0.53	0.15	0.03	0.3	1.0	<0.0001
FLAB vs. GBM	31	0.68	0.11	0.02	0.4	0.8	<0.0001
OA45% vs. OA2.5	31	0.40	0.16	0.03	0.2	0.9	<0.0001
OA45% vs. PH45%	31	**0.87**	0.10	0.02	0.5	1.0	<0.0001 ^a^
OA45% vs. GBM	31	0.70	0.12	0.02	0.4	0.8	<0.0001
OA2.5 vs. PH2.5	31	**0.90**	0.05	0.01	0.7	1.0	<0.0001 ^a^
OA2.5 vs. GBM	31	0.42	0.13	0.02	0.2	0.7	<0.0001
PH45% vs. PH2.5	31	0.40	0.15	0.03	0.2	0.9	<0.0001
PH45% vs. GBM	31	0.70	0.14	0.03	0.4	1.0	<0.0001
PH2.5 vs. GBM	31	0.42	0.12	0.02	0.2	0.7	<0.0001

a The spatial distribution of the metabolic tumor volumes was significantly different even if the segmentation algorithm was identical ($45\%{\text{SUV}}_{\text{max}}$ or SUV2.5), but applied by two different software products (Philips EBW4 or OASIS).

SD=standard deviation; SE=standard error; Min=minimum; Max=maximum.

### C. Impact of the DICOM transfer on the volumes

**Figure 3:**
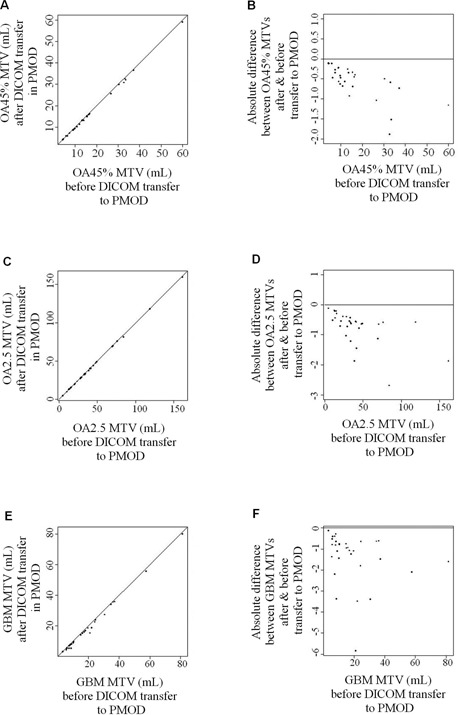
The scatter plots ((a), (c), (e)) show the metabolic tumor volumes (MTV) before the transfer to PMOD (x‐axis) plotted against the MTV (mL) after transfer in PMOD (y‐axis) for OASIS ((a): OA45%; (c): OA2.5) and for the gradient‐based method (e). The Bland‐Altman bias plots ((b), (d), (f)) show the difference between the volumes before the transfer to PMOD (x‐axis) against the absolute difference between the volume after the transfer in PMOD and the volume before transfer (y‐axis). The importation of the MTVs in PMOD significantly reduced the volumes delineated with OASIS software (OA2.5 & OA45%), and particularly the volumes delineated using the GBM for which the mean difference was superior to 1 mL (mean difference: ‐1.38mL±SD: 1.21 mL).

The transfer to PMOD significantly reduced the ${\text{GTV}}_{\text{CT}}$, the volumes delineated with OASIS software (OA2.5 and OA45%) and the gradient‐based volumes. It did not modify the volumes delineated with the Philips software (PH2.5 and PH45%) or FLAB. The volume changes are presented in Fig. 3. The Bland‐Altman plots show that the greater the MTV, the greater the difference in volume after the transfer in PMOD.

**Figure 4:**
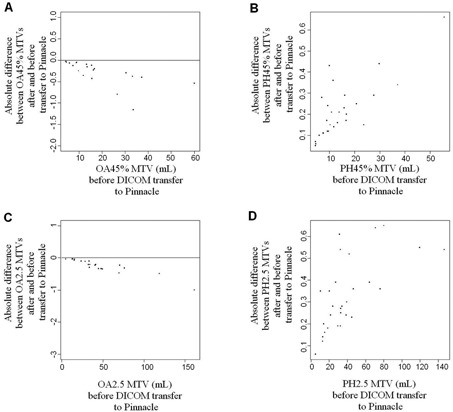
These Bland‐Altman bias plots show the difference between the metabolic tumor volumes (MTV) before the transfer to Pinnacle (x‐axis) against the absolute difference between the MTV (mL) after the transfer in Pinnacle and the volume before transfer (y‐axis). The transfer from OASIS ((a): OA45%; (c): OA2.5) to Pinnacle led to a reduction of the MTV, systematically. While the transfer from Philips EBW4 ((b): PH45%; (d): PH2.5) to Pinnacle systematically led to an expansion of the MTV.

The transfer of the MTVs to Pinnacle significantly changed the MTVs, whichever commercial algorithm was used (Fig. 4). The mean difference was ‐0.28±0.28ml(p=0.0002) and ‐0.25±0.22ml(p<0.0001) for the volumes delineated in OASIS with 45% and 2.5 thresholds, respectively. Conversely, the transfer from Philips EBW4 to Pinnacle systematically led to an expansion of the volume. The mean difference was 0.21±0.13ml(p<0.0001) and 0.33±0.16ml(p<0.0001) for the volumes delineated in Philips EBW4 with 45% and 2.5 thresholds, respectively.

## IV. DISCUSSION

The delineation of the MTV in oncology using PET is increasingly investigated in the literature. At diagnosis, MTV (along with the SUV derived from the MTV) may have a prognostic value; at the time of treatment planning, the MTV may be an additional tool for the delineation of the GTV and, finally during treatment, the MTV may be one of the parameters used for the estimation of the therapeutic response.^15^, ^16^, ^22^, ^23^, ^24^

Various segmentation algorithms are available, but none has been fully validated and widely implemented in the clinics. This is in part due to the absence of a gold standard, which ideally should be the measurements of corresponding pathological samples.^(25)^ Even these are imperfect, as tumor shrinkage may occur after formalin‐fixation, for example.^26^, ^27^

Obviously the segmentation algorithm has a major impact on the metabolic delineated volumes. Considering the absence of a recognized gold standard for assessing the tumor volume *in vivo*, our aim was to compare the MTVs to the clinical reference (i.e., the volume used for treating the patients, defined by a single radiation oncologist). Clearly, the ${\text{GTV}}_{\text{CT}}$ cannot be considered representative of the underlying truth, but it is the one used to treat the patients and the only reference at hand. The intraclass correlation coefficient between the MTVs and the ${\text{GTV}}_{\text{CT}}$ ranged from 0.26 to 0.85. Furthermore, the conformity index between the volume that was used for treating the patients (${\text{GTV}}_{\text{CT}})$ and the various MTVs ranged from 0.24 to 0.38, indicating major spatial variations in addition to changes in volumes. Quite surprisingly, the MTVs derived from the same threshold‐based method (e.g., SUV≥45% of the ${\text{SUV}}_{\text{max}})$ actually varied, depending on the commercial software that was used (i.e., Philips EBW4 or Segami OASIS). Such discrepancies are, in fact, easily explained by differences in implementation. For example, the default configuration of the Philips EBW4 does not draw the volume considering a percentage above the maximum pixel value but, in fact, above the average five pixels in the neighborhood of the maximum pixel value. We also observed that the MTV boundaries follow the tumor bordering voxels in Philips EBW4, while the contours in OASIS do not follow the edges of the voxels.

Quality assurance of each institutional PET system, patient preparation, PET images acquisition, and reconstruction parameters are known major factors affecting image quantification and subsequent segmentation.(^3,4^) Our work identified additional factors affecting the downstream MTV based on PET images. We found that the modification of the matrix of the CT and the subsequent resampling of the PET images resulted in a variation of the MTV from 0% to 142%; the increase of the extracted ${\text{SUV}}_{\text{max}}$ observed on resampled PET images on the Philips EBW4 is certainly related to the selection of five points (voxels or pixels) to average for the maximum SUV

**Figure 5:**
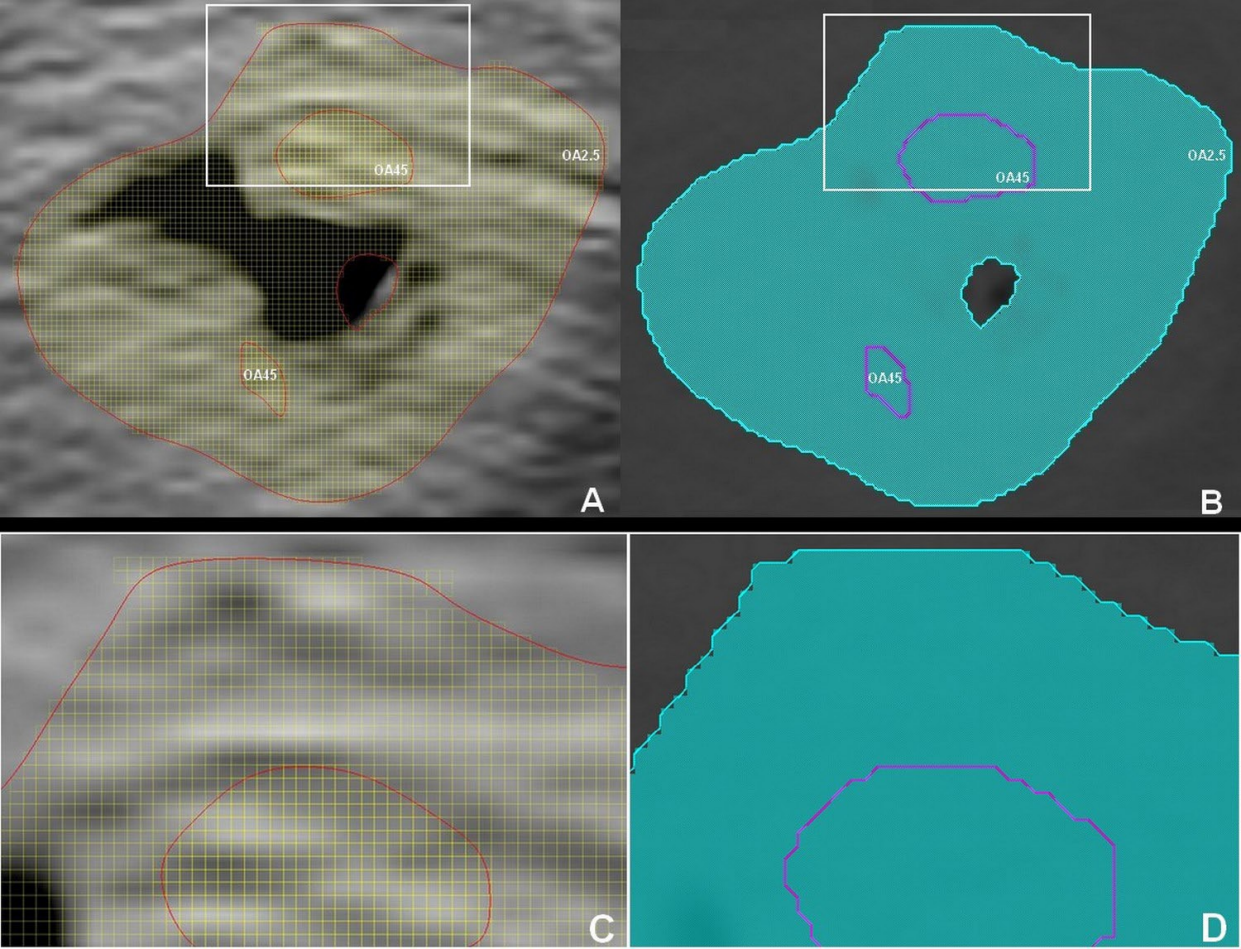
The transverse slice (a) shows the metabolic tumor volumes (MTVs) contours delineated in Segami OASIS software, using the relative threshold (OA45%) and an absolute threshold (OA2.5). The transverse slice (b) shows the MTVs transferred from Segami OASIS in Pinnacle. Magnified images (c) and (d) extracted from slices (a) and (b), respectively, show the slight difference between the contours delineated and displayed in OASIS (c) and contours displayed in Pinnacle (d) after the DICOM transfer.

One would expect the volumes to remain rigorously identical after export as DICOM/RT structures, yet we observed that some changes may occur in the process. Further, the algorithms are affected to variable extents, as the change in volume was statistically significant for OASIS and the GBM when transferred to PMOD, but not for the other methods. In OASIS, the volume boundaries crossed the frontline voxels, but the transfer to PMOD led to a reinterpretation of the boundaries following a specific algorithm implemented in PMOD and leading to subsequent volume changes. If the algorithm implemented follows the bordering voxels for one software and does not for the other one, then the transferred MTV will change. The larger the voxel size and/or the larger the MTV, the larger the volume modifications will be. Following the same principle, the DICOM transfer of a MTV from a commercially available PET workstation (Philips EBW4 or OASIS) to a commercially available treatment planning workstation (Pinnacle) led to significant changes in the MTVs (Fig. 5).

Both stage at diagnosis and tumor regression grade of the total mesorectal excision specimen independently determine patient survival.^(28)^ New therapy systems are, therefore, developed to deliver higher doses in a highly conformal manner to irregular target volumes. Considering the current treatment scheme of rectal cancer, the metabolic GTV, does not impact in a dramatic way the clinical target volume (CTV) that includes the GTV with 1 cm isotropic margin, the mesorectal subsite, and the pelvic lymph nodes at high risk of microscopic involvement.^(29)^ An additional 8 mm margin is then applied to the CTV to delineate the planning target volume (PTV) in order to minimize organ motion and setup errors. Some of the volume changes observed in this study, particularly those associated with the DICOM transfer, may be accounted for, especially in other tumor types for which the target volume delineation is more complex, such as lung cancer or head and neck carcinomas. These considerations also become of primary importance when highly conformal dose delivery systems are used, in particular intensity‐modulated radiation therapy (IMRT) and dose painting. More generally, it should be fully integrated as part of the quality assurance processing that is mandatory for any activity of radiation oncology.^(30^)

**Figure 6:**
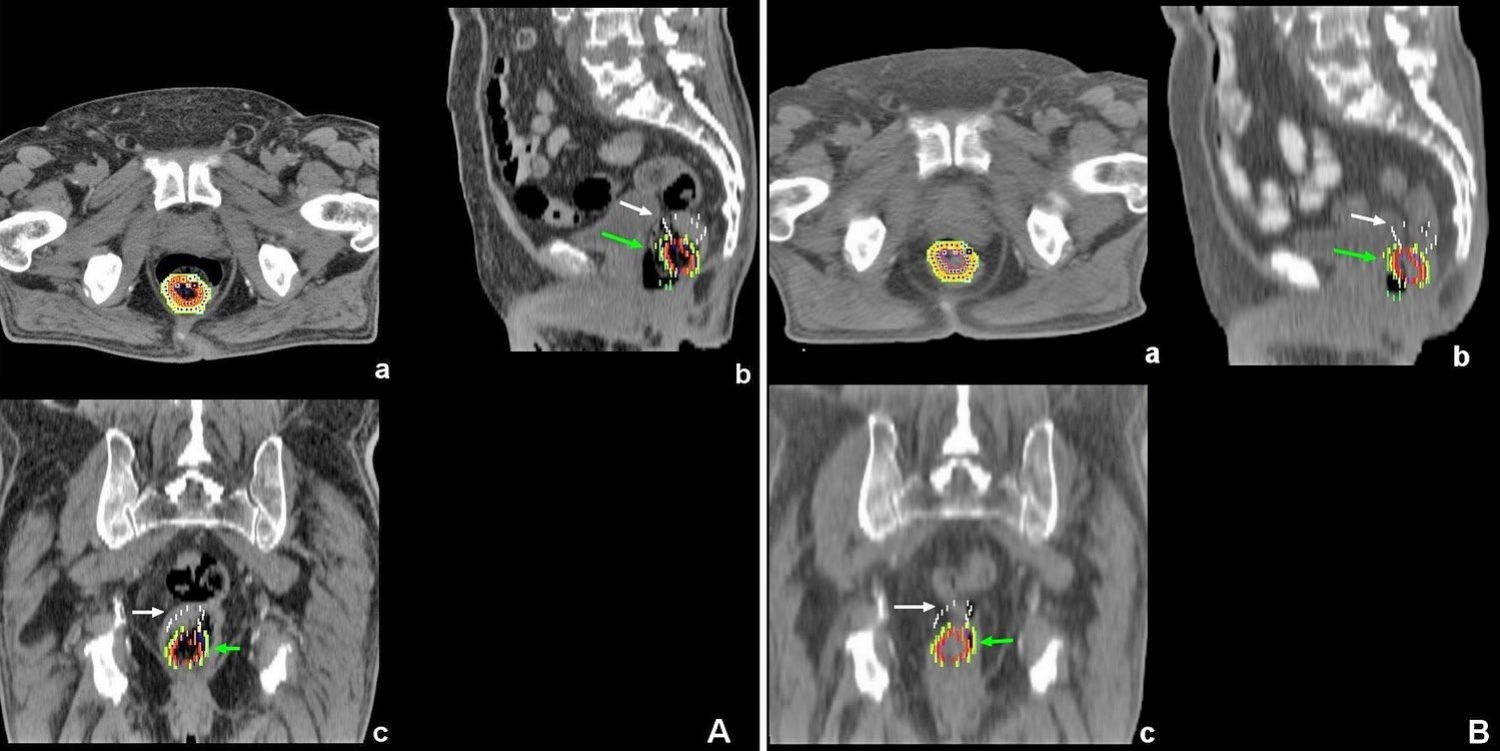
The figure shows an example of bowel and tumor displacement between the ${\text{CT}}_{\text{RT}}$ (left panels) and the CT of the FDG PET/CT (right panels; (a): transverse slice; (b): sagittal slice, and (c): coronal slice). Metabolic tumor volumes (FLAB=purple,OA45%=red; PH45%=orange; OA2.5=yellow; PH2.5=green) are indicated by the green arrow and the ${\text{GTV}}_{\text{RT}}$ (white contour) by the white arrow.

Our work presents several limitations. First, it lacks a true gold standard for defining the actual tumor volume, even though, as previously discussed, such gold standard does not exist. Phantom studies have been performed and showed differences in volumes depending on the algorithms, but the results cannot directly be translated into the clinical setting, as the complexity and the heterogeneity of clinical PET images are usually much greater than the available phantoms.^8^, ^9^, ^31^ In addition, organs and tissue are not completely immobile in the human body, and movements may occur during or between studies and lead to geographical misses (Fig. 6). In our work, the CI between the MTVs and the ${\text{GTV}}_{\text{RT}}$ was ≤0.38 and was 0 for four patients. Secondly, the point spread function (PSF) of our PET/CT system was only available for the $4\times 4\times 4\;{\text{mm}}^{3}$ voxel size, so that we were not able to perform the segmentation using the GBM on resampled PET images. The GBM has the advantage of being optimized for the individual PET/CT devices, but this requires measuring the PSF of the system. On the other hand, the FLAB algorithm requires less user intervention and is easier to implement, and appears to be highly robust without the need for calibration, at least as long as the parameters of the PET image are within a normal range usually encountered in clinical practice. The MTVs delineated with FLAB were not significantly affected either by the PET images resampling or by the transfer between the software products used in this study. Although this is clearly beyond the scope of this article, segmentation algorithms, such as FLAB or the GBM, should be preferred to methods based upon relative or fixed SUV thresholds, as they are more robust across a wide variety of intensity and heterogeneity of uptake.

## V. CONCLUSIONS

Distinct segmentation algorithms led to significantly different MTVs. Moreover, it was found that the MTVs are affected by the software used for implementing the segmentation algorithm, and that the manipulation of PET/CT images and MTVs, such as the DICOM transfer, may also induce additional volume variations. Furthermore, not all commercially available software products and segmentation algorithms are equally affected by these issues. It is, therefore, recommended to test these aspects before integrating the PET‐based volumes in the routine clinical radiation therapy planning, and to standardize all image processing and transfer procedures.

## ACKNOWLEDGMENTS

We thank Laurence Seidel and Professor A. Albert for providing statistical data. Part of this work was presented as a poster at the Annual Meeting of the Society of Nuclear Medicine, San Antonio, Texas, June 4–8, 2011.

## Supporting information

Supplementary Material

Supplementary Material
