# Supplementary figures and images for: FDG PET/CT for rectal carcinoma radiotherapy treatment planning: comparison of functional volume delineation algorithms and clinical challenges
Source: J Appl Clin Med Phys. 2014 Sep 8;15(5):216–28. doi: 10.1120/jacmp.v15i5.4696 (PMC5711099; doi:10.1120/jacmp.v15i5.4696)

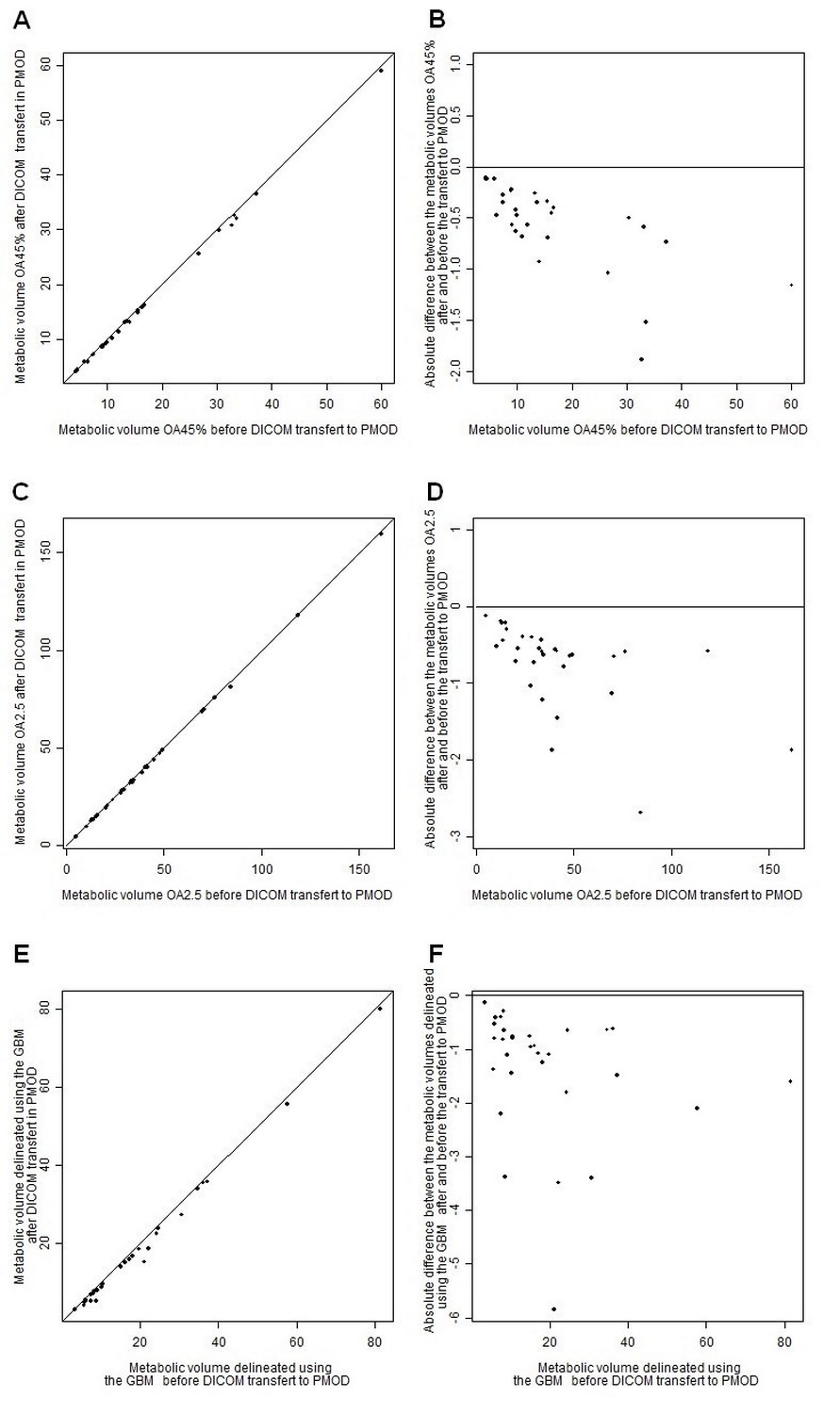

Supplement: Supplementary file 1 — Supplementary Material [file ACM2-15-216-s001.jpg]

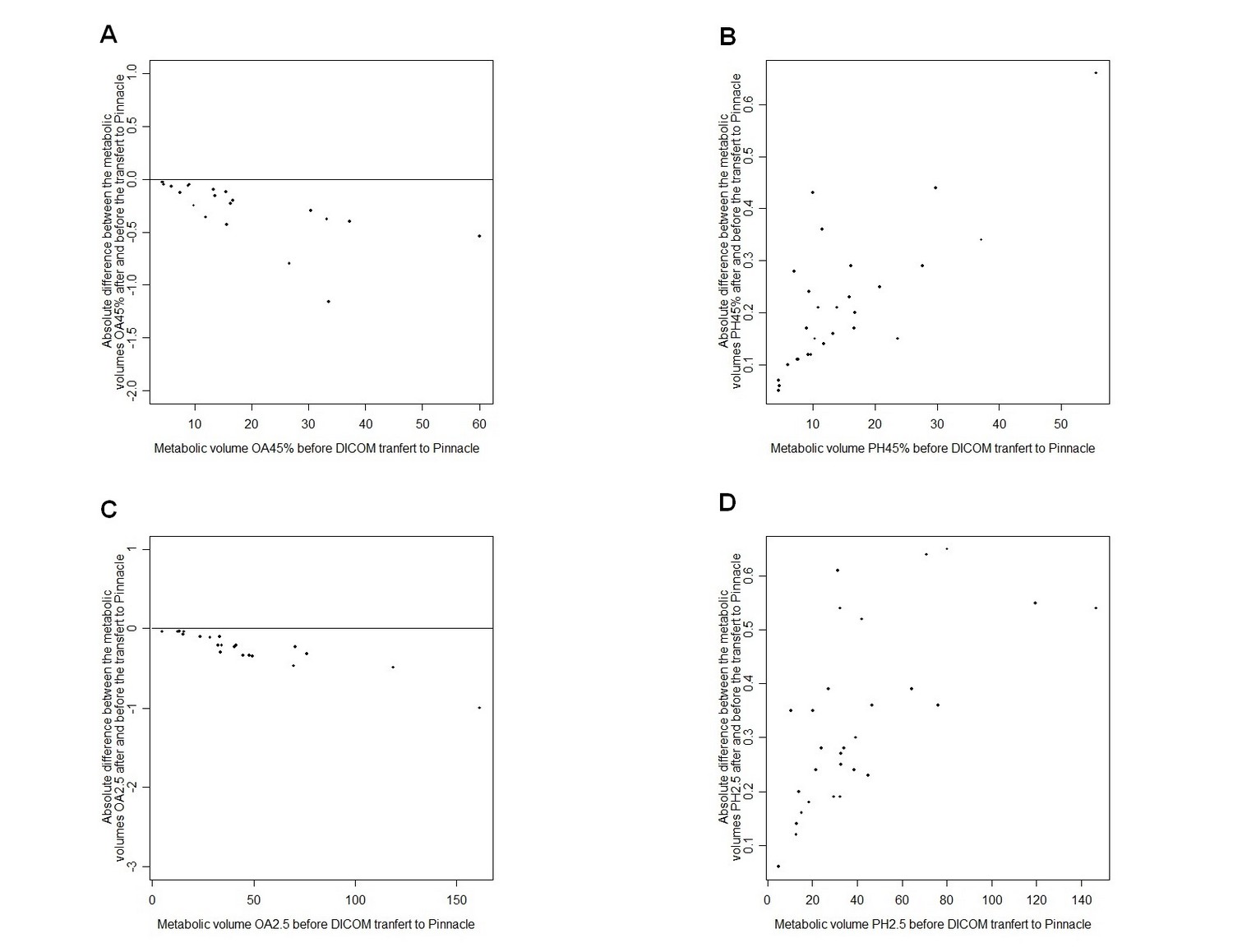

Supplement: Supplementary file 2 — Supplementary Material [file ACM2-15-216-s002.jpg]
